# Gut Microbiota and the Paradox of Cancer Immunotherapy

**DOI:** 10.3389/fimmu.2014.00157

**Published:** 2014-04-07

**Authors:** Theofilos Poutahidis, Markus Kleinewietfeld, Susan E. Erdman

**Affiliations:** ^1^Division of Comparative Medicine, Massachusetts Institute of Technology, Cambridge, MA, USA; ^2^Laboratory of Pathology, Faculty of Veterinary Medicine, Aristotle University of Thessaloniki, Thessaloniki, Greece; ^3^Departments of Neurology and Immunobiology, Yale School of Medicine, New Haven, CT, USA; ^4^Broad Institute, Massachusetts Institute of Technology and Harvard University, Cambridge, MA, USA; ^5^Faculty of Medicine, Dresden University of Technology (TUD), Dresden, Germany

**Keywords:** tumor macroenvironment, regulatory T-cells, cancer immunotherapy, inflammation and cancer, probiotic bacteria

## Abstract

It is recently shown that beneficial environmental microbes stimulate integrated immune and neuroendocrine factors throughout the body, consequently modulating regulatory T-lymphocyte phenotypes, maintaining systemic immune balance, and determining the fate of preneoplastic lesions toward regression while sustaining whole body good health. Stimulated by a gut microbiota-centric systemic homeostasis hypothesis, we set out to explore the influence of the gut microbiome to explain the paradoxical roles of regulatory T-lymphocytes in cancer development and growth. This paradigm shift places cancer prevention and treatment into a new broader context of holobiont engineering to cultivate a tumor-suppressive macroenvironment.

## Introduction

The neoplastic process is characterized by overwhelming complexity. Cancer is comprised of a genetically unstable population of cells that proliferate at an extraordinarily high rate. Millions of cancer deaths each year make it obvious that the battle against cancer is asymmetric, with humankind often being the weaker element (1). To date, cancer research efforts directly confront malignancy by targeting properties of individual cancer cells. In 2000, Hanahan and Weinberg described that most of the research on origins and treatment of cancer had just contributed toward “adding further layers of complexity to a scientific literature that is already complex almost beyond measure” (2).

In the same landmark paper, however, the authors were optimistic enough to predict groundbreaking upcoming advances in the conceptual rather than the technical level (2). They were proven right. One such advancement was the increased awareness for the importance of the tumor microenvironment in the etiopathogenesis of neoplasia (3, 4). We now know that initially transformed cells are much less autonomous in their growth than previously thought (5, 6). Among the microenvironment elements, immune cells and factors have emerged as fundamental players (4–6). Accumulating evidence suggests that tumor-associated inflammatory cell accumulation, whether overt or smoldering, could be viewed as a tumor-promoting event (7–9). These inflammatory responses enhance mutagenesis by oxidative DNA damage and shape the tumor stroma in favor of cancer cell survival and expansion (6, 10, 11).

Will this knowledge base in the field of inflammation, immunity, and cancer lead to new, highly effective, and biologically safe cancer immunotherapy modalities? We assert that the outcome will depend upon the philosophy and the strategic goals that will dominate the bench-to-bedside research. We propose that research in this field should focus upon stimulating systemic innate immune balance and adaptive immune resiliency, making the mammalian host more powerful to resist its cancer challenger. One possible approach utilizes gut microbiota or microbial antigens to stimulate beneficial immune cells. On the other hand, existing immunotherapy aims to selectively interrupt immune factors to better recognize and exterminate cancer cells (12–17), an approach that may ultimately lead to host instability. To further explain this point of view, we will refer to the recently discovered paradoxical roles of regulatory T-cells (T_REG_) in cancer (10, 14).

## T_REG_ are Central in Preserving Systemic Immune Homeostasis and Good Health

FOXP3^+^ CD4^+^ CD25^+/high^ T_REG_ are dominant cellular elements of the professional suppressor arm of the immune system and are important for orchestrating the control of peripheral immunological tolerance (18). The transcription factor FOXP3 is a fundamental regulator of T_REG_ function in rodents and humans, and so far the most reliable phenotypic indicator of their identity. Recent studies on human T_REG_ subpopulations, however, revealed that low but discernible levels of FOXP3 expression could be detected in non-suppressive T_REG_ or even in activated effector T-cells. It is probable that this finding reflects the inherent plasticity of T_REG_; FOXP3^+^ cells co-expressing effector T-cell phenotypic markers or cytokines may be in stages of a progressive, epigenetically regulated, phenotypical, and functional shift process (14, 16, 19–22), ultimately favorable for healthful recovery of the host after environmental challenges. The role of T_REG_ is central in preserving immune system homeostasis for health and the balance of beneficial inflammatory responses during infections while minimizing collateral tissue damage. In cancer, however, roles of T_REG_ are traditionally considered to be negative (14–16, 23).

## T_REG_ Gather Near Tumors and Favor Cancer Survival

A large body of data suggest that T_REG_ gather near tumors and suppress the anti-tumor inflammatory response, thus favoring cancer cell survival. To this end, tumor-associated T_REG_ are thought as a major impediment of anti-tumor vaccines (13–16, 23). Clinical and experimental data suggest that tumor-associated T_REG_ recognize both self and neoantigens expressed by tumor cells, counteracting antigen-specific effector T-cell responses. Consequently, immunotherapy strategies based on the vaccination with tumor-associated antigens fail to evoke an effective response against cancer cells due to the activation and expansion of tumor antigen-specific T_REG_ (14–16). This potential interplay of T_REG_ within tumors has been reviewed in detail elsewhere (12–16), and has led to the proposal of several anti-T_REG_ regimens for cancer immunotherapy. These regimens aim to deplete T_REG_, inhibit their suppressive function, prevent their homing into tumor sites, or block their differentiation/proliferation (12–16).

Several of these T_REG_-targeting modalities have already been tested in the clinic, with mixed results (13, 16). Blocking T_REG_ function by depleting the cytotoxic T-lymphocyte-associated antigen 4 (CTLA-4) appears promising (24), due to the depletion of T_REG_ from tumor tissues (25, 26). However, a similar regimen could lead to an opposite effect with the accumulation of T_REG_ and CD8^+^ T-cells in tumors (27, 28). A phase III study of melanoma patients using a gp100 peptide vaccine with interleukin (IL)-2 administration led to equally promising results with discovery of T_REG_ expansion in responding patients (29).

## Gut Microbiota Induce Potent T_REG_ with Systemic Anti-Neoplastic Properties

As the results of these trials are anticipated, the literature reveals contradictory evidence. Indeed, the studies associating high densities of tumor-associated cells expressing T_REG_ markers including FOXP3 with a poor prognosis in several types of human cancers are now challenged by similar studies on the very same types of cancer showing the opposite outcome (30–34). The different CD8^+^:T_REG_ ratios and the presence of FOXP3^+^ cell subsets of undetermined identity in the tumor microenvironment have been proposed as probable explanations (16). Indeed, data from animal models show under certain conditions of microbial priming that T_REG_ not only protect but also alter the tumor microenvironment to induce remission of already established intestinal, mammary, and prostate cancers (35–41). The hypothesis that the composition of the different subsets of FOXP3^+^, which may include effector Foxp3^+^ cells, is intriguing (16). Indeed, it was previously shown that IFN-γ levels were increased during T_REG_-mediated tumor regression in mice (37). Further, feeding of probiotic microbes to mice induces systemic oxytocin secretion that shifts immunity toward IFN-γ and CD25 for improved wound healing capacity and systemic good health (42). A question subsequently arising is whether gut microbiota may be engineered to harness an anti-neoplastic FOXP3^+^ cell milieu (5, 10, 41).

## Gut-Centric Hypothesis: Prior Exposures to Microbes Explain Beneficial Roles of T_REG_

Stimulated by a gut-centric systemic homeostasis hypothesis, we set out to explore and explain the paradoxical roles of T_REG_ in cancer using several different mouse models of cancer and adoptive cell transfer methodologies (10). We found that T_REG_ may suppress, promote, or have no effect in carcinogenesis depending upon their timing and prior exposure to gut bacterial antigens and presence of IL-10 (35–39, 41, 43, 44). Under some conditions, adoptive transfer of T_REG_ rapidly led to apoptosis of emerging tumor cells (37, 45). Using as a model organism an opportunistic pathogen, *Helicobacter hepaticus*, commonly residing in the lower bowel of mice, we have shown in Rag2-deficient mice (otherwise lacking lymphocytes) that gut microbiota modulate inflammatory bowel disease and inflammation-associated colon cancer, a cancer process inhibited by properly functioning IL-10-dependent T_REG_ (35, 36). Subsequently, by introducing *H. hepaticus* into the large bowel flora of mice lacking the APC tumor suppressor gene (*Apc^Min/^*^+^), we found that intestinal polypogenesis was greatly enhanced by bacteria and subsequently suppressed by immune-competent T_REG_. Furthermore, adenomas of infected *Apc^Min/^*^+^ mice progressed into adenocarcinoma, a transition atypical of polyps of aged-matched uninfected controls (38, 41). Interestingly, *Apc^Min/^*^+^ mice having *H. hepaticus* in their gut flora were prone to develop cancer in tissues distant from intestine, such as prostate and the mammary glands (40, 41, 43, 46, 47). *H. hepaticus*-induced tumorigenic events were inhibited by supplementation with T_REG_ from immune-competent wild type donor mice.

**Figure 1:**
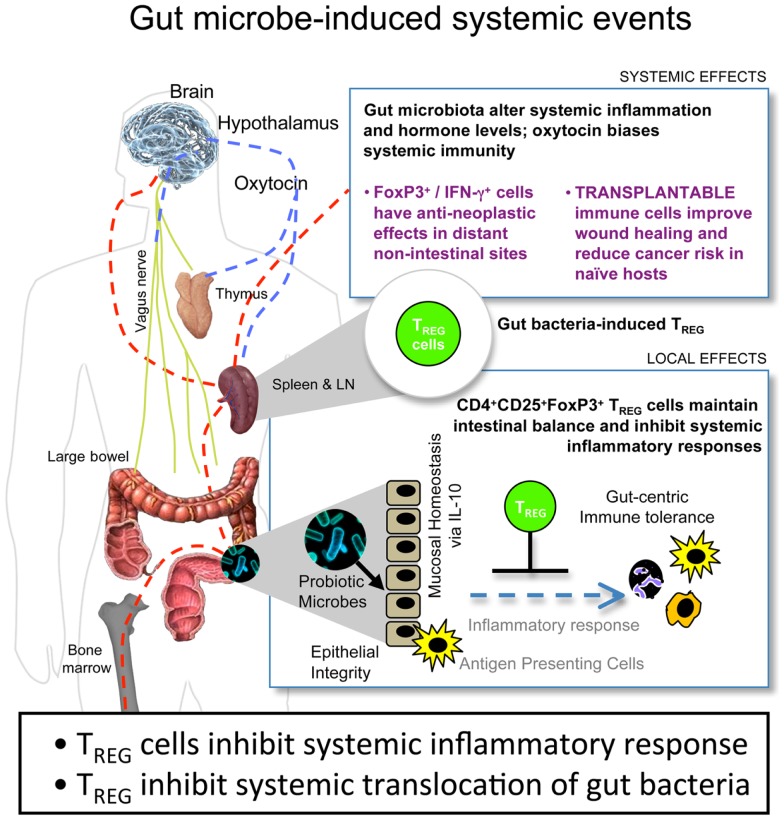
**Gut bacteria–host crosstalk is continuous and reciprocal in the cancer macroenvironment**. Beneficial microbes trigger IL-10-mediated GI-tract immune and neuronal networks that lower systemic inflammatory tone and up-regulate hypothalamic–hypophyseal targets, including oxytocin, constituting a gut–systemic immunity-endocrine-axis. In this way, microbiota stimulate CD4^+^ lymphocytes including regulatory T cells (T_REG_) that suppress, promote, or have no effect in carcinogenesis depending upon their timing and prior exposure to gut bacterial antigens and presence of interleukin (IL)-10. This places neoplastic development and growth into a new broader context of the holobiont (comprised of the mammalian host plus resident microbes) and the cancer macroenvironment, highlighting microbes that may be engineered for sustained good health.

A potent treatment to counteract these local and systemic *H. hepaticus*-induced tumorigenic events was supplementation with T_REG_ in an IL-10-dependent manner (10, 36, 38–40, 44, 46, 48). Purified T_REG_ exhibited greatest anti-cancer potency when taken from donor mice previously colonized with *H. hepaticus*. By contrast, T_REG_ taken from donor mice without prior *H. hepaticus* exposure were ineffective, and in some cases actually enhanced tumorigenesis (10). Based on these results, we theorize that the tumor microenvironment is subject to systemic inflammatory events arising from environmental exposures in the gastrointestinal tract (Figure 1). This microbe-inducible pro-inflammatory condition contributes to tumor trophic signaling. Interestingly, bacterial antigen triggered IL-10-dependent activities in the GI-tract impart sustained protection from the aforementioned events, resulting in immune cell recruitment, including T_REG,_ which, by being more potent in their anti-inflammatory roles, work locally and systemically to suppress sepsis, myeloid precursor mobilization, and inflammatory signaling important in extra-intestinal cancer evolution (10, 43). These systemic events comprise the tumor macroenvironment.

The roles of intestinal microflora in promoting cancer development within the bowel have been well established (35, 49–52). Linking gut microbial flora and local and systemic effects that promote (38) or suppress (45) tumors throughout the body, expands this paradigm in a challenging manner. Recent findings show that gut flora imbalances considerably undermine the response to both immune (53, 54) and non-immune chemotherapeutic regimens, such as cisplatin and oxaliplatin (53).

## A Weakened T_REG_ Feedback Loop Unifies Autoimmune Diseases and Cancer

These gut microbe-centric findings in mice are consistent with the “hygiene hypothesis,” according to which insufficient microbial exposures earlier in life predispose to allergies, autoimmune disorders, and uncontrollable inflammation-associated pathologies later in life. We have shown that the basic principles of this hypothesis may apply not only to auto-immunity, but also to neoplastic disease as well, and that T_REG_ play a central role in this phenomenon (10, 41, 55). The ability of T_REG_ to decrease risk for cancer and counteract established tumors depends upon microbe-triggered IL-10, which works to maintain immune system homeostasis and reinforce a protective anti-inflammatory, anti-neoplastic T_REG_ phenotype (41). T_REG_ display inherent phenotypic plasticity (10). Hygienic individuals with a weakened IL-10 and T_REG_ feedback loop are prone to a re-direction of unstable resting peripheral T_REG_ toward a T helper (Th)-17 pro-inflammatory process. As a result “hygienic” subjects are at higher risk to develop auto-immune diseases and cancer (10). It is tempting to postulate that this may explain why only a few people go on to develop cancer, while nearly everyone bears dysplastic and early neoplastic lesions throughout their body (56).

Depending on composition of gut microbiota, the immune system of mice may acquire different subclinical characteristics, even in the absence of overt inflammatory processes. The clinically silent immune system status may determine the risk of developing sporadic cancer in epithelia throughout the body. Further, we found that consuming beneficial probiotic bacteria led to the expansion of a Foxp3^+^ cell population in the periphery (42, 45, 57) conferring protection to diet-related and genetic predisposition to mammary cancer (45). Targeted oral challenge with such probiotic bacteria resulted in the activation of interrelated systemic inflammatory and metabolic pathways, either through blood circulation or via the vagus nerve (Figure 1). Consequently, there was an upregulation of systemic hormone levels, such as oxytocin, testosterone, and thyroxin. Oxytocin serves to sustain immune and integumentary homeostasis, biasing the immune system toward IL-10 and IFN-γ, without anergy, subsequently minimizing the deleterious systemic effects of IL-17 (57). This altered immune system and metabolic profile of mice imparted healthful phenotypes including shiny fur and youthful hair follicle cycling, accelerated skin wound healing capacity, and resistance to diet-induced obesity and senility (42, 47, 57, 58). Through tightly regulated immune activities, competent T_REG_ permit brief beneficial host inflammatory responses to eliminate invading pathogens, and later inhibit chronic deleterious inflammatory tissue damage (43). The results of our wound healing assays further suggest that the probiotic microbe-induced enhancement of the T_REG_-dominated arm of the immune system did not compromise the ability of mice to respond to invading pathogens (42).

## Beneficial Systemic Effects of Gut Microbes are Transplantable via Foxp3^+^ T_REG_ into Naïve Hosts

Adoptive cell transfer models offer mechanistic insight as these beneficial effects were isolated to bacteria-primed T_REG_ (42, 47, 57–59). In fact, healthful phenotypes were entirely reproducible in naive recipient mice by the adoptive transfer of highly purified T_REG_ derived from probiotic-fed cell donors (42, 57, 59). These results suggest gut microbe-induced crosstalk with the host in a continuous and reciprocal manner. The fate of preneoplastic and neoplastic lesions arising in epithelia throughout the body depends upon this macroenvironment at the whole organism level. Consequently, the tumor macroenvironment is defined as the “holobiont,” i.e., the mammalian organism plus the microbial symbionts it bears. The T_REG_ population is a central player of the tumor macroenvironment connecting gut bacteria with reproductive fitness, youthful phenotypes, and anti-neoplastic properties.

## Microbial Engineering Offers New Strategies for Public Health

Taken together, microbial engineering strategies using food-grade bacteria highlight alternative directions in cancer immunotherapy. Modulating beneficial T_REG_ via diet is a biologically safe and efficient approach, originating from genetic programs that have been shaped during the millions of years of co-evolution of mammals with their gut bacteria symbionts. These attributes remain largely inactive in individuals with a modern lifestyle, Westernized dietary habits, and stringent hygiene practices. Awakening these latent T_REG_-mediated capabilities may provide an alternative avenue to reduce cancer risk at a population level for public health. The perspectives presented here should be considered as an alternative paradigm – not only for fighting cancer – but also for promoting overall good health and longevity.

## Author Contributions

Theofilos Poutahidis, Markus Kleinewietfeld, and Susan E. Erdman wrote the paper.

## Conflict of Interest Statement

The authors declare that the research was conducted in the absence of any commercial or financial relationships that could be construed as a potential conflict of interest.
